# Noise Removal with Maintained Spatial Resolution in Raman Images of Cells Exposed to Submicron Polystyrene Particles

**DOI:** 10.3390/nano6050083

**Published:** 2016-04-29

**Authors:** Linnea Ahlinder, Susanne Wiklund Lindström, Christian Lejon, Paul Geladi, Lars Österlund

**Affiliations:** 1 Swedish Defence Research Agency, FOI, Cementvägen 20, SE-901 82 Umeå, Sweden; christian.lejon@foi.se; 2 Department of Forest Biomaterials and Technology, Swedish University of Agricultural Sciences, SE-901 83 Umeå, Sweden; paul.geladi@slu.se; 3 Department of Engineering Sciences, The Ångström Laboratory, Uppsala University, P.O. Box 534, SE-751 21 Uppsala, Sweden; lars.osterlund@angstrom.uu.se

**Keywords:** Raman spectroscopy, Raman imaging, super-resolution, particles, cells, Tikhonov regularization

## Abstract

The biodistribution of 300 nm polystyrene particles in A549 lung epithelial cells has been studied with confocal Raman spectroscopy. This is a label-free method in which particles and cells can be imaged without using dyes or fluorescent labels. The main drawback with Raman imaging is the comparatively low spatial resolution, which is aggravated in heterogeneous systems such as biological samples, which in addition often require long measurement times because of their weak Raman signal. Long measurement times may however induce laser-induced damage. In this study we use a super-resolution algorithm with Tikhonov regularization, intended to improve the image quality without demanding an increased number of collected pixels. Images of cells exposed to polystyrene particles have been acquired with two different step lengths, *i.e.*, the distance between pixels, and compared to each other and to corresponding images treated with the super-resolution algorithm. It is shown that the resolution after application of super-resolution algorithms is not significantly improved compared to the theoretical limit for optical microscopy. However, to reduce noise and artefacts in the hyperspectral Raman images while maintaining the spatial resolution, we show that it is advantageous to use short mapping step lengths and super-resolution algorithms with appropriate regularization. The proposed methodology should be generally applicable for Raman imaging of biological samples and other photo-sensitive samples.

## 1. Introduction

Confocal Raman spectroscopy is an analytical method which has demonstrated great utility in a wide range of applications in life science applications, such as cancer diagnostics [1] and—as exemplified in this study—detection of particles inside cells [2,3,4,5,6,7]. Some advantages are that the method requires minimal sample preparation, is label free and non-destructive [2,3,4,5,6,7,8,9,10,11]. Furthermore, it is possible to collect hyperspectral images of the sample by step-wise measurements of small sample volumes [4,5,6,7,8,10,11]. Each pixel in such a (hyperspectral) Raman image contains a Raman spectrum with chemical information from molecules and crystals with Raman active vibrational modes [2,3,4,5,6,7,9]. It is possible to simultaneously detect particles inside cells [2,3,4,5,6,7] and to use the spectral information to discern organelles without using dyes, fluorescent labels, or microtome sectioning and associated embedding methods [4,5,6,7,8,9,10,11]. Besides providing a better understanding of the particle toxicity [2,3,4,5], Raman images of cells exposed to nanoparticles can be useful in the development of nanomaterials for drug delivery applications and diagnostics [6,7].

A limitation with confocal Raman spectroscopy is the spatial resolution, which is low compared to the resolution typically obtained with electron microscopy, the routine method to visualize particles in cells [8,12]. The spatial resolution is related to the diameter, *d_o_*, of the laser spot, approximated by Abbe diffraction limit as:

(1) $$d_{0}\approx \frac{1.22\lambda }{NA}$$

where λ is the laser wavelength and *NA* is the numerical aperture of the microscope objective [13]. With λ = 514 nm and *NA* = 0.9, *d_o_* ≈ 700 nm. Formally the diffraction limit is defined by the Airy disk [13]. The Rayleigh criterion states that two objects can be resolved if they are separated by the radius of the Airy disk. According to the Rayleigh criterion the lateral spatial resolution is about 350 nm for λ = 514 nm and *NA* = 0.9 [13]. The spatial resolution in confocal Raman microscopes is however worse in transparent samples, especially in the depth direction, since the laser can penetrate deep into the sample and give contribution from off-focal plane scattering centers [13]. Also, the number of pixels and the distance between them, *i.e.*, the step length, affect the spatial resolution [14,15]. The best spatial resolution is achieved if pixels are collected with step lengths shorter than the diameter of the laser-illuminated volume [14,15]. With motorized *X*-*Y* tables, it is possible to collect pixels with about 100 nm spacing, and with piezoelectric controlled devices nm step lengths are readily achieved. Because of the measurement time it is, however, most often necessary to find a compromise between spatial resolution and spectral quality, in particular for light-sensitive samples [16].

A human lung epithelial cell is typically about 20 µm in diameter. Raman mapping of cells with 100 nm step length would hence generate images that contain *ca.* 40,000 pixels. There are reports of cell damage after irradiation with a 514 nm laser after a few minutes [16], although we have previously shown that measurement times around 1 h are possible [3,5]. The measurement time per pixel is therefore in practice limited to less than 1 s, which is a very short measurement time to detect few, small particles or to acquire a high-quality spectrum of the fingerprint region where many Raman bands assigned to DNA, proteins, lipids and carbohydrates are present [9].

A complementary approach to improve the image quality, rather than modifying acquisition conditions, is to use super-resolution algorithms, which either combine the information from multiple, low-resolution images, shifted relative to each other, or alternatively estimate a super-resolution image from a single, low resolution image with pixels that contain information from overlapping measurement volumes [17,18,19,20,21,22]. Super-resolution algorithms were originally developed for analysis of photographic images, but have also successfully been used to improve the resolution of hyperspectral images [17,18,19,20,21,22]. Super-resolution Raman spectroscopy has, however, so far been limited to silicon-based samples [17,18,19] and atmospheric, metal-based particles [17], and has not previously been applied to images of biological samples.

In the present paper, super-resolution is for the first time applied to Raman images of biological samples, *i.e.*, cells exposed to submicron particles, which are difficult to image with good spatial resolution since they are transparent, give a comparatively low Raman signal and also have a limited measurement time because of the cell’s sensitivity to the laser irradiation. Images acquired with two different step lengths (100 nm and 500 nm) are compared, and are also compared to their corresponding super-resolution images.

## 2. Results and Discussion

### 2.1. Principal Component Analysis

Raman images of human lung epithelial cells exposed to polystyrene submicron (PS) particles were collected with two different step lengths: 100 nm (referred to as the “100 nm image”) and 500 nm (referred to as the “500 nm image”). Principal component analysis (PCA) was used to visualize particles and cells in the images, and also to reduce the number of variables (wavenumbers) prior to applying super-resolution algorithms, by selecting the first 2 principal components.

In PCA, the variance in data is summarized by new, orthogonal variables, so called principal components (PCs) [8,10]. The first PC describes the largest variance [8,10]. All spectra/pixels in the Raman images are given score values, which are interpreted as concentrations of the corresponding PC [8,10]. The loadings are the weights that should be multiplied with the original variables, *i.e.*, wavenumbers, in order to get the score values [8,10]. They should be interpreted together with the scores [8,10]. It should however be noted that negative loading values do not imply that the original spectra have peaks with negative values [8,10].

**Figure 1:**
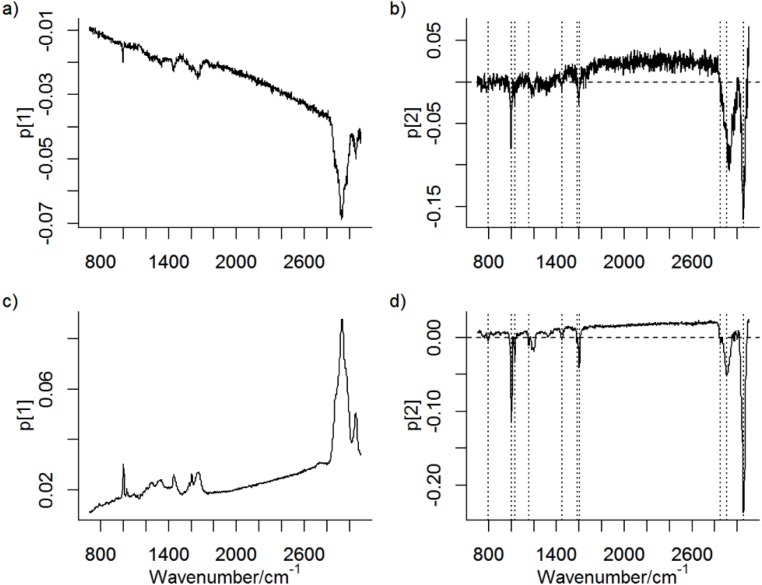
Loadings for principal components (PCs) ((**a**) PC1 and (**b**) PC2) for principal component analysis (PCA) of the 500 nm image. Loadings for (**c**) PC1 and (**d**) PC2 for PCA of the 100 nm image. Raman bands assigned to polystyrene submicron (PS) particles are marked with dashed lines.

Figure 1 shows the loadings of the first PC (PC1) and second PC (PC2) from PCA models of the 100 nm image and the 500 nm image. It is evident that PC1 captures the fluorescence background and an overall intensity variation in the Raman spectra with pronounced contribution from the Raman band at ≈2900 cm^−1^ due to ν(C–H) stretching modes, which can be used to visualize the cells in Raman images [6,7,11]. In the 500 nm image, the lowest intensities and lowest score values are observed for the pixels acquired from the cell and the highest intensity and highest score values are observed from pixels from outside of the cell. An opposite response is observed in PC1 for the 100 nm image.

**Figure 2:**
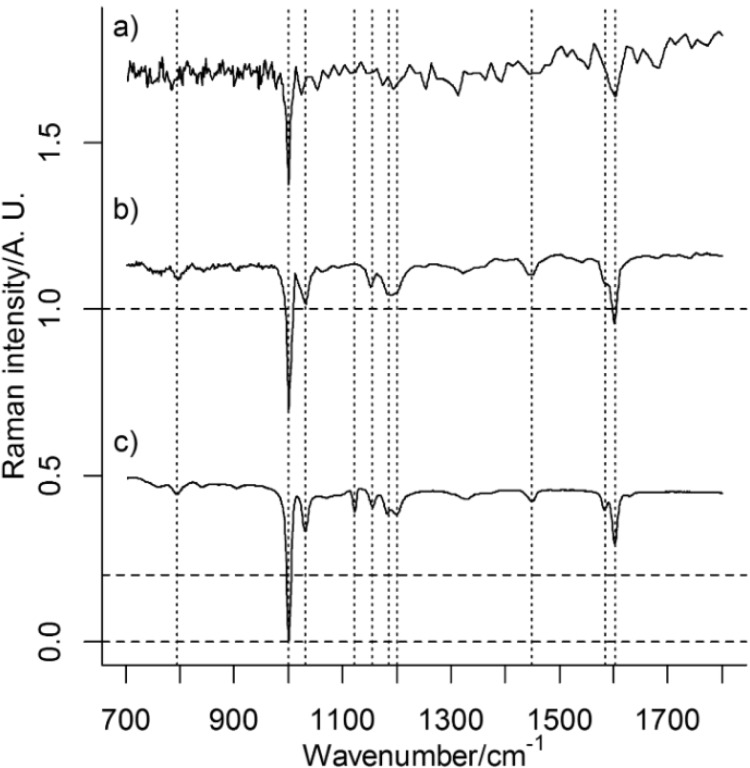
Loadings for PC2 for (**a**) the 500 nm image; and (**b**) the 100 nm image. (**c**) Reference spectrum of PS multiplied by −1. Raman bands assigned to PS are marked with dashed lines. An intensity offset have been added to the spectra.

**Figure 3:**
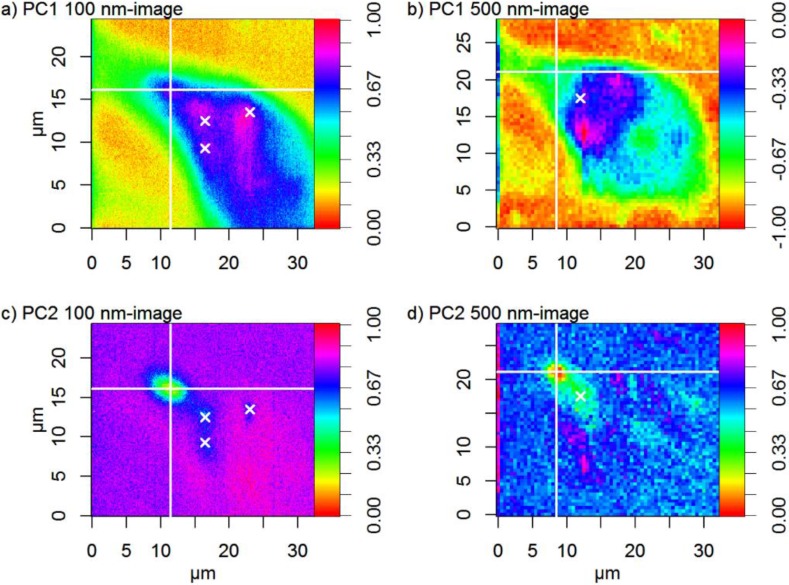
Normalized score maps of PC1 from the PCA model of (**a**) the 100 nm image and (**b**) the 500 nm image. Normalized score maps of PC2 from the PCA of (**c**) the 100 nm image and (**d**) the 500 nm image. The large crosses (lines at *X* ≈ 11.5 µm and *Y* ≈ 16.1 µm in the 100 nm image and at *X* ≈ 8.5 µm and *Y* ≈ 21 µm in the 500 nm image) and small crosses show the geometrical centers of large and small particle agglomerates, respectively. Score values in image b) have been multiplied with −1 for easier comparison. The maps are slightly offset by ≈ 3 µm (*X*) and ≈ 5 µm (*Y*) and the (0,0) position is thus not the same in the 100 nm image and the 500 nm image. The 100 nm image covers a mapping area of 24.4 × 32.5 µm^2^, and the 500 nm image 28.5 × 32.5 µm^2^.

**Figure 4:**
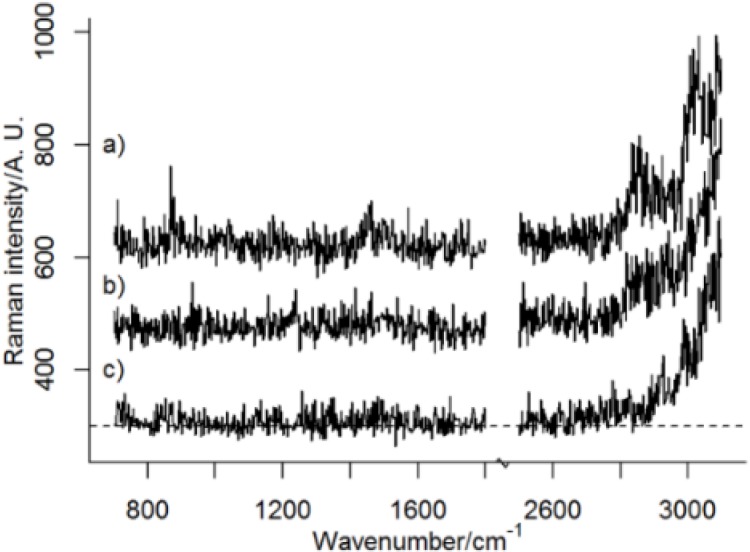
Raw spectra from (**a**) a pixel centered at the largest particle agglomerate; (**b**) a pixel from the cell in a region free from particles; and (**c**) the background (substrate). All pixels are from the 100 nm image. An intensity offset have been added to the spectra.

PS is in both PCA models described by PC2, whose loading plots have similarities to a reference spectrum of PS (Figure 2). In both models, pixels/spectra from PS have low score values in PC2 (apparent as negative bands in Figure 2b,d). Variables that are correlated may be described by the same PC, irrespective of the origin of their variance. It is therefore possible that PC2 also contains spectral information from other sources than PS. However, the presence of characteristic Raman bands from PS, their relative intensities, which agrees with the relative intensities of the strongest Raman bands from PS (Figure 2), and the score maps (Figure 3b,c), which show probable particles, suggest that PC2 represents PS very well. It is worth noting that the spectral quality is very low in single pixel spectra (Figure 4) and multivariate analysis such as PCA, which considers averaging over many pixels, can therefore be considered as more reliable than an analysis of single Raman bands. Raw spectra showed only small variations in fluorescence background. Spectral pre-treatment, such as baseline correction, was therefore not necessary. The small variation in fluorescence background seems also to be explained by PC1 and not PC2 (see Figure 2).

It can be seen that the loading plots for the PCA of the 500 nm image are noisier than the corresponding loading plots for the PCA of the 100 nm image. This is because of the comparatively few spectra in the 500 nm image: 3,705 pixels compared to 79,300 pixels in the 100 nm image. It should be mentioned that the measurement time per pixel is the same in both images, which means that the signal to noise ratio per pixel can be considered as equal in both cases. The total measurement time of the 500 nm image is thus much shorter than the total measurement time of the 100 nm image. A question to be answered is if images acquired with long step lengths, *i.e.*, short measurement times, can be sufficiently improved by super-resolution algorithms such that similar information can be extracted from these images as from images acquired with a shorter step length.

**Figure 5:**
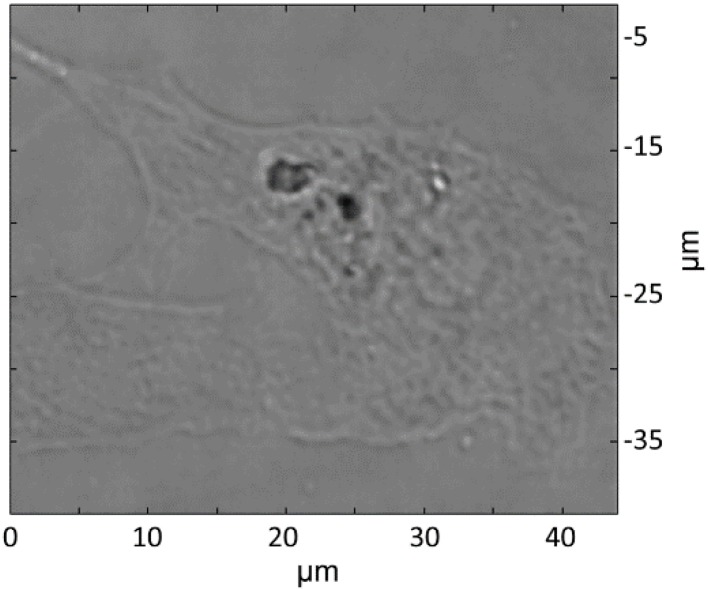
Optical microscope image of the Raman mapped cell. The cell has been exposed to PS particles.

**Figure 6:**
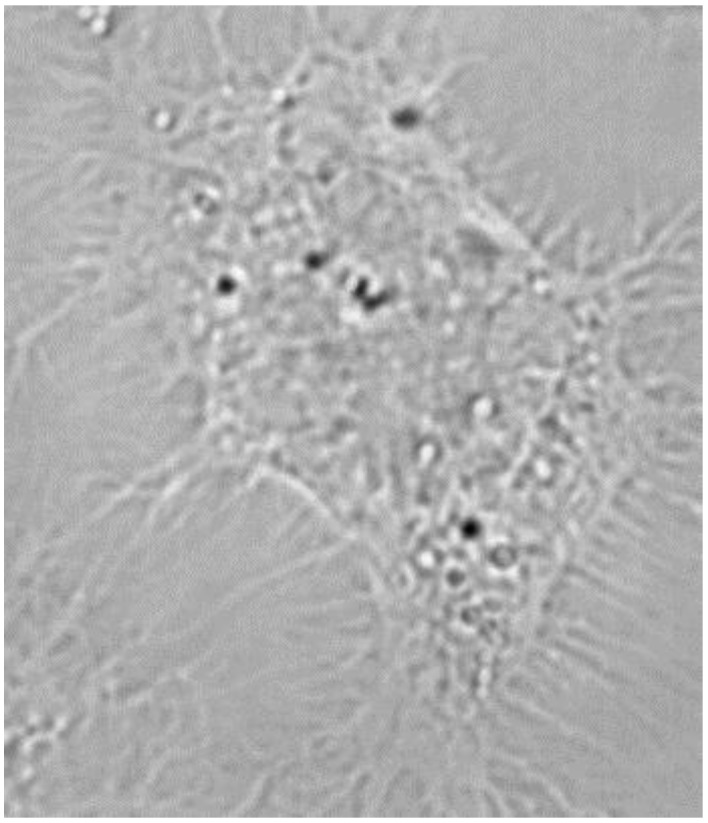
Optical microscope image of a cell that has not been exposed to PS. Black dots are normally visible inside cells although they have not been exposed to PS.

Figure 5 shows the Raman mapped lung epithelial cell. Large particle agglomerates can be observed directly in the optical microscope image, but this image alone is not enough to prove that particles are inside the cell. As a comparison, Figure 6 shows an optical microscope image of a control cell, which was not exposed to particles. Typically, black dots, which do not represent PS particles, are visible inside cells in optical microscope images of unexposed cells. Thus inspections of optical microscopy images alone cannot be used to distinguish PS particles in cells.

In contrast, the score maps in Figure 3 show that PS particles are located inside cells. Figure 3c,d shows the position of PS particles and Figure 3a,b shows the cell. By overlaying Figure 3a with Figure 3c and Figure 3b with Figure 3d, it is evident that PS particles are located inside the cell. The position of the largest PS particle agglomerate is at *X* ≈ 11.5 µm and *Y* ≈ 16.1 µm in the 100 nm image and at *X* ≈ 8.5 µm and *Y* ≈ 21 µm in the 500 nm image. Some smaller particle agglomerates are located to the right and below the larger agglomerate, and are centered at *X* ≈ 16.5 µm (100 nm image) and *X* ≈ 11.5 µm (500 nm image). These smaller particle agglomerates and the larger particle agglomerate are not well-separated in the 500 nm image. The particle agglomerate seen at *X* ≈ 23 µm, *Y* ≈ 13.5 µm in the 100 nm image is also difficult to discern in the 500 nm image, where the background is noisier than in the 100 nm image. Most of the particle agglomerates seen in the score images are much larger than the primary particles, which have a diameter of 300 nm. It is well-known that particles in biological matrices tend to agglomerate and that they are surrounded by proteins [23]. It is thus expected that particles in a biological system are much larger than corresponding primary particles.

### 2.2. Super-Resolution and Effect of Regularization Parameter

Score maps were treated with a super-resolution algorithm to remove noise. A general assumption in super-resolution algorithms is that measured, low resolution images (here: score maps) are blurry, noisy, warped and decimated versions of a super-resolution image, X [17,18,19,20,21,22]. For *N* low resolution images ${\left\{Y_{i}\right\}}_{i=1}^{N}$, the relationship can be expressed as:

(2) $$Y_{i}=D_{i}W_{i}B_{i}X+E_{i}$$

where $D_{i}$ denotes decimation, $W_{i}$ denotes the geometric warp, $B_{i}$ corresponds to the blur function, X is the super-resolution image, and $E_{i}$, is the noise in image *i*, respectively [17,18,19,20,21,22]. Since the data was collected in a single mapping experiment, the step length is at high-resolution whereas the optical resolution is low. In our algorithm, the sequence of low-resolution images is constructed by combining subsets of pixels from single mapping acquisitions. The geometric warp and the decimation are thus known. The blur, *i.e.*, the unresolved details, is here represented by the point spread function (PSF), *i.e.*, the response of the imaging system to a point source [17,18,19,20,21,22].

X can hence be estimated from $Y_{i}$, the PSF ($B_{i}$), the geometric warp, $W_{i}$, and the decimation, $D_{i}$, by minimizing the sum of squared residuals [17,18,19,20,21,22]:

(3) $$\text{min}({∥D_{i}W_{i}B_{i}X-Y_{i}∥}^{2})$$

The minimum norm solution in Equation (3) is an ill-posed problem, which means that the solution is very sensitive to noise [17,18,19,20,21,22]. To find a robust solution it is therefore necessary to use regularization, which means that a regularization term is added to suppress noise. We have used Tikhonov regularization to calculate the magnitude of *X*:

(4) $$\text{min}({∥D_{i}W_{i}B_{i}X-Y_{i}∥}^{2}+{∥\alpha X∥}^{2})$$

Different forms of regularization terms are possible and Tikhonov prioritize keeping a small gradient in the image, and so here the regularization parameter, α, is used to adjust the smoothing of the super-resolution image, X [17,18,19,20,21,22]. In essence, super-resolution is built into the mathematical formulation posing it to an ill-posed problem, and the Tikhonov choice of regularization term creates a trade-off between sharp contrast and noise removal. It is thus crucial to choose an α-value that gives enough noise removal and at the same time does not alter important information in $Y_{i}$.

Detailed descriptions of the super-resolution concept and (Tikhonov) regularization are beyond the scope of this article. We refer the reader to other publications for details of these methods [17,18,19,20,21,22].

**Figure 7:**
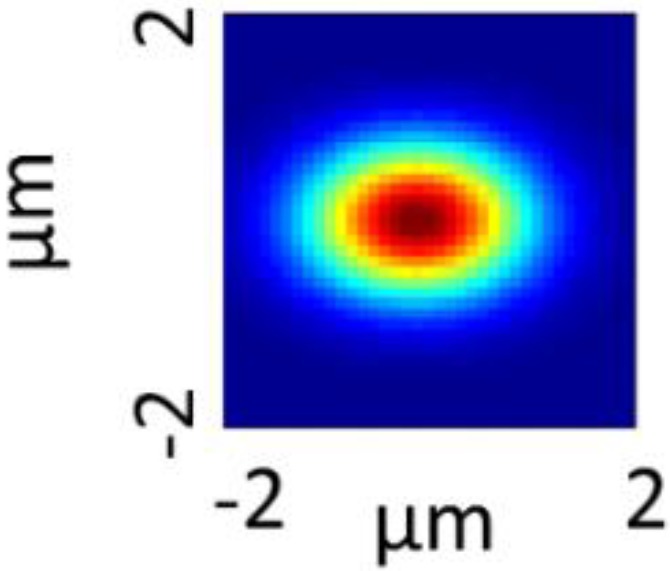
Point spread function (PSF) approximated by fitting Gaussian functions to the derivatives of line maps from measurements across the edge of an Au structure in a 1951 USAF patterned Au/Si reference sample.

The first derivative of intensity profiles, *i.e.*, line mappings, over the border of a sharp edge between known materials can be used as an approximation of the PSF [17]. Here we measured across the edge of an Au structure in a 1951 USAF patterned Au/Si reference sample to determine the PSF. The PSF (Figure 7) was approximated from the measurements of the 1951 USAF patterned Au/Si reference sample by fitting Gaussian functions to the derivatives of the Si Raman band at 520.7 cm^−1^ intensity profiles. The experimentally determined PSF map was found to be slightly elliptic, similar to previous reports using same types of Raman microscopes [17]. The full width at half maximum (FWHM) of the PSF was 1.22 µm (Y-direction) and 1.70 µm (*X*-direction). This is much larger than the theoretical spatial resolution according to the Rayleigh criterion (350 nm). It should be remarked that the contrast in the Raman image of the 1951 USAF patterned Au/Si reference sample is much higher than the contrast in the Raman images of particles in cells. The approximated PSF can therefore be expected to be much narrower than the true PSF in the Raman images of cells. The approximated PSF from the Au/Si reference was used as a reasonable approximation since there are no sharp edges in the Raman images of cells from where a reliable PSF can be approximated.

**Figure 8:**
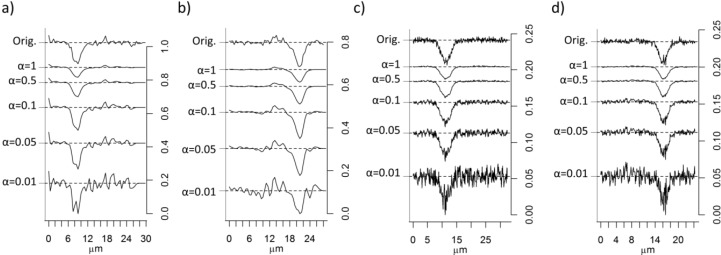
Intensity line scans of score-values from thePC2 score maps in Figure 3c,d after application of the super-resolution algorithm with different α-values at different location in the images where particles are located: (**a**) *X* = 8.5 µm in PC2 for the 500 nm image; (**b**) *Y* = 21 µm in the 500 nm image; (**c**) *X* = 11.5 µm, in the 100 nm image; and (**d**) *Y* = 16.1 µm in the 100 nm image. The curves are shifted along the ordinate axis for clarity.

The effect of different α-values was studied in score maps of PC2. Images were treated with the super-resolution algorithm with α varying between 0.01 and 1, and the intensity profiles of the lines at *X* = 11.5 µm, *Y* = 16.1 µm in the 100 nm image and at *X* = 8.5 µm and *Y* = 21 µm in the 500 nm image were compared (Figure 8). All line maps are centered at the largest particle agglomerate. We can expect small α-values to give very unstable solutions to Equation (4) because of its ill-conditioned nature. Images generated with α-values that are too low will thus contain noise and artefacts. This is clearly seen in Figure 8, where it is shown that α = 0.01 actually gives images with more noise than the original images. α-values, that are too high, on the other hand, produce too much smoothing and thus a loss of important information. The maximum and minimum derivatives of the PC2 line scans are shown in Table 1. Hard regularization removes high frequency components, such as noise, but also sharp edges. This is illustrated in Table 1, where it can be seen that the derivatives are impaired with higher α-values. The derivatives of the peak in the intensity profiles are actually worsen in images calculated with high α-values compared to the derivatives in the original score maps. In order to improve the derivatives α = 0.05 is found to be a suitable α-value while maintaining reasonable smoothness in the image. α = 0.01 gives even sharper transition between the particle and the cell in the 100 nm image, but the noise level is evidently increased. To achieve a clear noise removal, it seems, however, that a much higher α-value, α = 0.5, is necessary. Images where thus generated with both α = 0.05 and α = 0.5 to represent enhanced resolution and smoothed images respectively.

The spatial resolution improvement upon application of super-resolution can be assessed by studying the edge of PS intensity lines. Analogous to the FWHM calculation for the PSF determination, edge contrast of PS particles were found by calculating FWHM of the *derivative* of the intensity lines at both the positive and negative side. Table 2 summarizes the calculated FWHM for the original images and the images generated with α = 0.05 and α = 0.5. It is seen that FWHM is not decreased, compared to the original images, by applying super-resolution algorithms except for the resolution in the *X*-direction in the 500 nm image, α = 0.05. It can also be noticed that the resolution is similar in the *X* direction compared with the line mappings of Au on Si (the 1951 USAF patterned test sample measured to estimate the PSF). In the *Y*-direction on the other hand the PS sample has lower edge contrast, which shows the inhomogeneity of the particle agglomerates and possibly resolution “artefacts” of transparent samples [13]. Table 2 suggests that the resolution is maintained upon application of super-resolution algorithms and the main effect of the super-resolution algorithm to our images is evidently removal of noise.

### 2.3. Comparison of Super-Resolution Images

**Figure 9:**
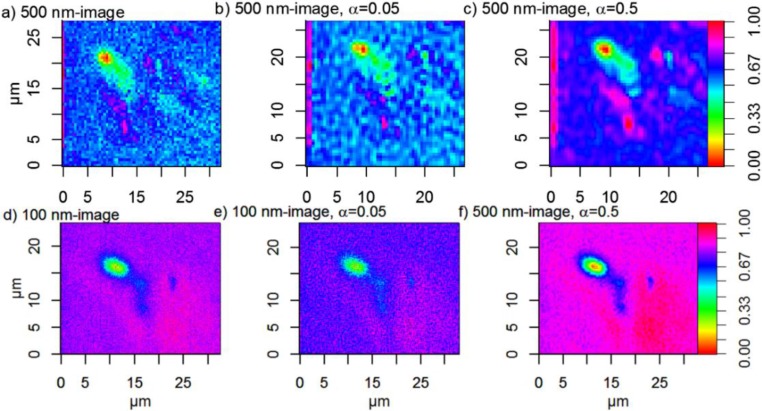
Normalized images of PC2, *i.e.*, score values ranging from 0 to 1. (**a**) 500 nm image; (**b**) 500 nm image treated with super-resolution algorithm, α = 0.05; (**c**) 500 nm image treated with super-resolution, α = 0.5; (**d**) 100 nm image; (**e**) 100 nm image treated with super-resolution algorithm α = 0.05; (**f**) 100 nm image treated with super-resolution algorithm, α = 0.5. Note that the (0,0) point is not the same in the 500 nm images and the 100 nm images.

The original and super-resolution-treated images of PC2 are shown in Figure 9. The super-resolution-treated images are slightly smaller than the input images. This is because of the procedure where low-resolution images are formed from the score maps by picking out a subset of pixels. This procedure gives too few pixels with overlapping measurement volumes at the edges of the images to estimate a noise-reduced image by using the super-resolution algorithm.

The smoothing effect of α is evident in Figure 9. The images generated with α = 0.5 contain much less noise and are smoother than the original images as well as the images generated with α = 0.05. The suspected particles are also easier to discern from the background because of the reduced noise and an increased contrast. The *image quality* is however *not* improved with α = 0.05. The 500 nm image treated with the super-resolution algorithm α = 0.05 has actually a noisier background than the original 500 nm image. The 100 nm image treated with the super-resolution algorithm and α = 0.05 has a lower contrast than the original image. It is evident that harder regularization than α = 0.05 is necessary to improve the image quality.

**Figure 10:**
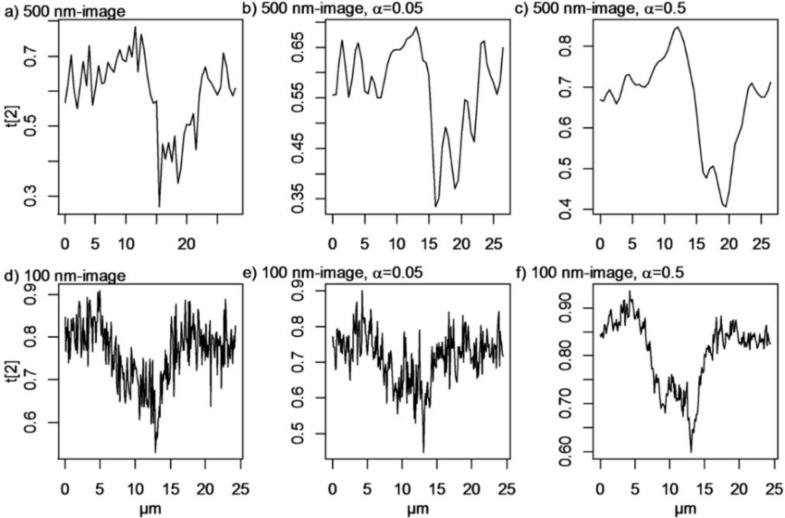
Intensity profiles for lines at X = 16.5 µm from the PC2 score (t[2]) map from the (**a**) 500 nm image; (**b**) super-resolution 500 nm image, α = 0.05; and (**c**) super-resolution 500 nm image, α = 0.5 Intensity profiles at *X* = 11.5 µm from the PC2 score (t[2]) map of the (**d**) 100 nm image; (**e**) super-resolution 100 nm image, α = 0.05; and (**f**) super-resolution 100 nm image, α = 0.5.

Intensity profiles for line mappings at *X* ≈ 16.5 (100 nm image) and *X* ≈ 11.5 (500 nm image), *i.e.*, lines centered over the smaller particle agglomerates, were compared to test how well particles positioned next to each other can be separated. The intensity profiles are shown in Figure 10. The exact number of particles and their diameters are unknown, since it is well-known that particles in biological matrices often are agglomerated and have proteins adsorbed to their surfaces [23]. A fit of the line mappings to the “true” size of the particles is therefore complicated and a precise answer to the number of closely positioned particles that can be separated before and after application of super-resolution algorithms is hence difficult to achieve. Figure 10a–c shows broad peaks centered at *ca.* 20 µm in the 500 nm images, while Figure 10d–f shows broad peaks centered at about 10 μm with diffuse shoulders at about 7 μm in the 100 nm images. The intensity profile for the line scan of the original 500 nm image appears to have one broad intensity maxima. Super-resolution with α = 0.05 has only a slightly smoothing effect, and amplifies artefact noise structures (Figure 10c). Two intensity maxima can possibly also be discerned in the super-resolution 500 nm image with α = 0.5. The penalty in noise reduction is, however, worse spatial resolution. The noise is not reduced for the 100 nm image when applying super-resolution with α = 0.05. The amplitude of the peak is however decreased, and it is thus even more difficult to discern particles in this image. Clear improvements of the 100 nm image are however seen when super-resolution is applied with α = 0.5 (Figure 10f). The noise is strongly reduced and the two apparent intensity maxima suggest that the line mapping is centered over two large particle agglomerates. Our results show that application of super-resolution algorithm is meaningful only when the sampling step-size is smaller than the object size and that in such cases considerable improvement of noise reduction can be achieved with maintained spatial resolution.

**Figure 11:**
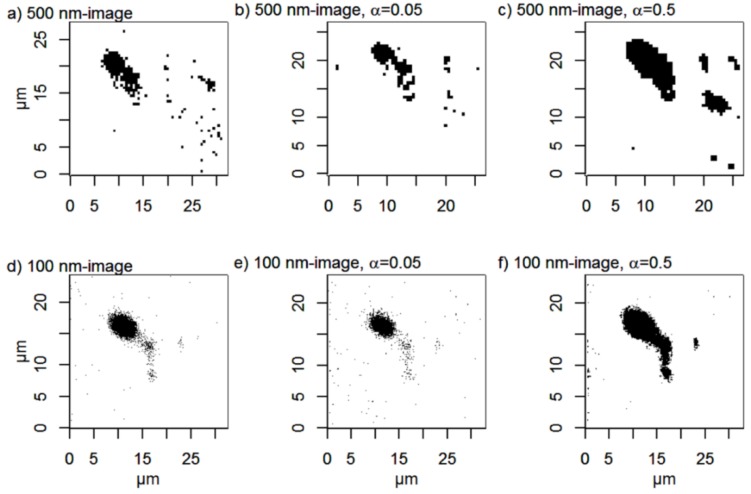
Threshold images of the score map for PC2 of (**a**) 500 nm image; (**b**) super-resolution 500 nm image, α = 0.05; (**c**) super-resolution 500 nm image, α = 0.5; (**d**) 100 nm image; (**e**) super-resolution 100 nm image, α = 0.05; (**f**) super-resolution 100 nm image, α = 0.5. The arrows point at a particle agglomerate that is difficult to detect without applying super-resolution. Threshold values are calculated with the “triangle method” [24].

Threshold images (Figure 11) were constructed from the score maps to study how many particles and agglomerates that can be detected in the images. The information is also summarized in Table 3, which shows the number and sizes of detected particle agglomerates (≥0.09 µm^2^) in the threshold images.

The original 500 nm image is noisy and several single pixels are classified as particles. These particles are not visible in the 100 nm image and they can therefore be regarded as suspected false positives. Super-resolution removes many of these suspected false positives, while other pixels, which are not classified as particles in the original image, become more pronounced with increasing α. In the super-resolution 500 nm image with α = 0.5, four large particle agglomerates are detected, while only two of them are visible in the original 500 nm image. This emphasizes the importance of using a regularization parameter that suppresses noise. It is important to identify possible artefacts in the original images by using e.g., threshold images and line scans before applying super-resolution and to compare the result of regularization with several α-values. Possible artefacts introduced by using insufficient regularization may otherwise give false positives (see Figure 10c). Taken together this suggests that super-resolution algorithms do not improve the overall quality of our images acquired with long step lengths (500 nm).

In contrast, improvements are clearly seen in the 100 nm image. The smoothing effect of α is also evident. With a small α-value, few, small, particles, or noise, are detected, while a high α-value gives a smoother image, where few, large particles are seen. Only regularization with α = 0.5 enhances the contrast compared to the original image, and the small suspected particle located at *X* ≈ 23 µm, *Y* ≈ 13.5 µm is now much easier to discern from the background than in the original 100 nm image. The effect of super-resolution α = 0.05 is very small in the 100 nm image.

In conclusion, our results show that application of super-resolution algorithms does not significantly improve the spatial resolution beyond the theoretical microscopy limit in our biological samples (Figure 8, Figure 9, Figure 10 and Figure 11). Instead we find that the major advantage of applying super-resolution in these particular applications is noise removal with *maintained* spatial resolution, which facilitates reliable detection of particles in complex biological matrices, as visualized by intensity lines over closely positioned particles and by threshold images, where small particles, smaller than can be distinguished from noise in the original images, can be observed. The smoothing effect of the Tikhonov regularization is adjusted by the regularization parameter α: high α-values give smoother images, but may on the other hand give less sharp edges. In this case, the original images are noisy and it is therefore advantageous to use a high α-value in order to detect more particles in the images. Images calculated with super-resolution algorithms using high α-values yield improved contrasts and reduced noise, which facilitates the separation of closely positioned particle agglomerates. The effect is most pronounced in the 100 nm step-size images compared to the 500-nm step-size images acquired with the same measurement time per pixel, which is not surprising since it contains many more pixels than the 500 nm image. The original 500 nm image contains more noise than the 100 nm image and some of the artefacts are enhanced after application of the super-resolution algorithm. To detect as many particles as possible and at the same time avoid noise, it is therefore suggested to collect images with a short step length and thereafter apply super-resolution algorithms to further improve the image quality.

While others have demonstrated image quality enhancements in Raman images by application of super-resolution algorithms in e.g., Si-based materials [18] and atmospheric inorganic particles [17], this is the first time a super-resolution algorithm is applied to Raman images of more complex, biological samples. In contrast to Si-based samples, the different components of a Raman image of a cell exposed to particles, e.g., the cell itself or the particles inside the cell, cannot be assessed by integration of a single Raman band, and multivariate techniques are therefore required. In contrast to more stable samples, such as the Si-based samples and particle samples, it is not possible to have long integration times to collect the images of cells, because of the risk of photo damage. We have here demonstrated that the super-resolution algorithm improves the image quality and maintains the image resolution of Raman images in biological samples. Enhanced image quality is of the utmost importance because of the limited measurement time in such samples.

## 3. Materials and Methods

### 3.1. Samples and Sample Preparation

Commercially available PS in water (Sigma-Aldrich, Buchs, Switzerland) with specified diameter 300 nm was used in the experiments. The hydrodynamic diameter was measured to be 332.2 ± 5.3 nm using photon cross correlation spectroscopy (Nanophox, Sympatec, Clausthal-Zellerfeld, Germany).

A 1951 USAF patterned test sample made of 30 nm thick Au deposited on a Si wafer was measured to determine the PSF. The pattern consists of groups of Au lines with widths ranging from 0.55 µm to 2000 µm. The test sample was prepared by electron-beam lithography.

Cell samples were prepared by culturing A549 cells on CaF_2_ substrates in cell media (RPMI-1640, 10% fetal calf serum, 50 mg·mL^−1^ gentamicin) at 37 °C in a humidified atmosphere with 5% CO_2_. The cells were allowed to attach overnight, and were thereafter exposed to 10 μg·mL^−1^ PS in phosphate buffered saline (PBS) for 24 h. Cells were fixated with 5 mL methanol, washed 2 times with PBS and placed in a Petri dish of the same before measurements. Only cells with characteristic morphology, as determined by visual inspection, were used in the Raman experiments.

### 3.2. Raman Spectroscopy

Raman spectra were collected with a Horiba JobinYvon HR 800 confocal Raman spectroscope (Horiba, Villeneuve d’Ascq, France) equipped with a Newton EMCDD detector (Andor, Belfast, UK), a 514 nm Ar ion laser (yielding about 6.5 mW on the sample) and a 60× water immersion objective (*NA* = 0.9). The confocal hole was 200 µm in diameter in all experiments. A 300 groove·mm^−1^ grating was used for measurements of cells, while a 600 groove·mm^−1^ grating was used for the measurements of the 1951 USAF patterned test sample that was measured to determine the PSF. The step length was 100 nm or 500 nm using a motorized XY-table (cells) or 100 nm using the DuoScan system (1951 USAF patterned test sample), *i.e.*, the laser beam, instead of the sample stage, is moved by using piezo-controlled mirrors. The measurement time was 0.1 s per pixel in all mapping experiments. Calibration against the 520.7 cm^−1^ Raman band in Si was performed before measurements.

### 3.3. Data Analysis

PCA on mean centered data in the 700 cm^−1^ to 3100 cm^−1^ spectral region was used to reduce the number of variables (1600 wavenumbers) to 2 PC:s that summarize the largest variance in data.

The regularization parameter, α, was varied between 0.01 and 1 as described in the Results and Discussions section. Besides α, it is also necessary to specify how many pixels in the original image that should be picked out to form low-resolution images from which the super-resolution images are estimated. The number of pixels used to form the low-resolution images, which is also the size of the region where the PSF is defined, was set to 6 (100 nm image) or 13 (500 nm image). This corresponds to 49 and 196 low resolution-images, respectively. Details of the super-resolution concept and Tikhonov regularization can be found elsewhere [17,18,19,20,21,22].

PCA and super-resolution calculations were performed in MatLab Ver. 7.10 (Mathworks, Natick, MA, USA). Images were generated and analyzed in R 3.2.0 (R development core team, R foundation for statistical computing, Vienna, Austria). Thresholds in the threshold images were set with the triangle thresholding method [24] in ImageJ 1.44p (U.S. National Institutes of Health, Bethesda, MD, USA). This is a geometric method that assumes a maximum mode close to the end of the histogram of gray scale levels.

## 4. Conclusions

Cells exposed to 300 nm polystyrene particles were imaged by spatially resolved Raman spectroscopy employing short (100 nm) and a long (500 nm) pixel step-size. A super-resolution algorithm that uses Tikhonov regularization was used to reduce noise in images of score maps from a PCA of spatially resolved Raman spectra. Score maps and super-resolution-treated images show cells and particle agglomerates inside cells. The images have been compared via comparison of the intensity profiles of line mappings over particle agglomerates visible in the images and comparison of threshold images.

It is shown that in order to reliably detect as many particles as possible and at the same time avoid noise, it is advantageous to map the sample with a short step length and to apply super-resolution. Super-resolution was not found to improve the spatial resolution (yielding clearly resolved ≈1.4 µm features in cells) for the cell samples used here. The spatial resolution was however maintained and the noise level was significantly reduced, which facilitated unambiguous identification of particles and makes it possible to separate closely positioned particles.

Super-resolution algorithms are here suggested as methods that can be used to improve the quality of images of samples that are sensitive to the laser irradiation and long measurement times and are therefore difficult to map with both high spectral quality and high image quality.

In general, we conclude that application of PCA and super-resolution based on images with shorter step size than the Rayleigh resolution (here 100/350 ≈ 0.3), with appropriate choice of regularization, provides a means to readily identify particles in cells that is not possible in optical microscopy (see Figure 6), from raw Raman images, or univariate analysis of Raman images.

## Figures and Tables

**Table 1:** Maximum and minimum derivatives at the inflection points of the peak in the intensity profiles in Figure 8.

Image	α-value	Max. Deriv.	Min. Deriv.	Image	α-value	Max. Deriv.	Min.Deriv.
500 nm	Orig.	X: 3.0 × 10^−3^	X: −2.5 × 10^−3^	100 nm	Orig.	X: 1.3 × 10^−4^	X: −1.5 × 10^−4^
		Y: 2.7 × 10^−3^	Y: −2.8 × 10^−3^			Y: 1.1 × 10^−4^	Y: −1.1 × 10^−4^
	α = 0.01	X: 3.6 × 10^−3^	X: −2.4 × 10^−3^		α = 0.01	X: **1.6 × 10^−4^**	X: **−1.9 × 10^−4^**
		Y: 3.0 × 10^−3^	Y: −3.5 × 10^−3^			Y: **1.2 × 10^−4^**	Y: **−1.3 × 10^−4^**
	α = 0.05	X: **3.8 × 10^−3^**	X: **−2.7 × 10^−3^**		α = 0.05	X: 1.4 × 10^−4^	X: −1.6 × 10^−4^
		Y: **3.2 × 10^−3^**	Y: **−3.5 × 10^−3^**			Y: 1.1 × 10^−4^	Y: −1.1 × 10^−4^
	α = 0.1	X: 3.6 × 10^−3^	X: **−2.7 × 10^−3^**		α = 0.1	X: 1.4 × 10^−4^	X: −1.5 × 10^−4^
		Y: 3.0 × 10^−3^	Y: −3.3 × 10^−3^			Y: 1.0 × 10^−4^	Y: −1.1 × 10^−4^
	α = 0.5	X: 2.1 × 10^−3^	X: −1.7 × 10^−3^		α = 0.5	X: 1.0 × 10^−4^	X: −1.1 × 10^−4^
		Y: 1.9 × 10^−3^	Y: −2.0 × 10^−3^			Y: 8.0 × 10^−5^	Y: −8.0 × 10^−5^
	α = 1	X: 1.4 × 10^−3^	X: −1.2 × 10^−3^		α = 1	X: 8.2 × 10^−5^	X: 9.0 × 10^−5^
		Y: 1.3 × 10^−3^	Y: −1.4 × 10^−3^			Y: 6.2 × 10^−5^	Y: −6.2 × 10^−5^

**Table 2:** Average value of full width at half maximum (FWHM) obtained from the derivatives of the intensity lines in Figure 8 (original image and α = 0.05 and α = 0.5) and the measurements across the edge of a Au structure in a 1951 USAF patterned Au/Si reference sample. n.a.: not applicable.

Image	Mean FWHM (µm)		
	Original Image	α = 0.05	α = 0.5
1951 USAF patterned Au/Si reference sample.	X: 1.70	n.a.	n.a.
	Y: 1.22		
500 nm image	X: 1.71	X: 1.42	X: 1.91
	Y: 2.14	Y: 2.40	Y: 2.23
100 nm image	X: 1.68	X: 1.78	X: 1.78
	Y: 2.52	Y: 2.58	Y: 2.55

**Table 3:** Summary of the number of particle agglomerates (≥0.09 µm^2^) in the threshold images in Figure 11.

Image	Particle Size (µm^2^)	Number of Particles.	Image	Particle Size (µm^2^)	Number of Particles.
500 nm image	0.25	22	100 nm image		
	0.5	4		0.18	1
	0.75	3		1.72	1
	4	1		18.84	1
	32.5	1			
500 nm image, α = 0.05	0.25	7	100 nm image, α = 0.05		
	0.5	1			
	0.75	1		0.09	2
	1	1		0.13	3
	1.25	1		0.16	2
	1.75	1		14.89	1
	21.75	1			
500 nm image, α = 0.5	0.25	2	100 nm image, α = 0.5	0.11	1
	1	2		0.13	1
	2.5	1		0.16	1
	3.5	1		0.22	1
	12.25	1		0.25	1
	50.75	1		1.35	1
				46.66	1

## Abbreviations

The following abbreviations are used in this manuscript:

NA	Numerical aperture
FWHM	Full width at half maximum
PBS	Phosphate buffered saline
PC	Principal component
PCA	Principal component analysis
PS	Polystyrene
PSF	Point spread function
